# Polymorphism of fucosyltransferase 3 gene is associated with inflammatory bowel disease: a systematic review

**DOI:** 10.2478/abm-2023-0044

**Published:** 2023-09-17

**Authors:** Jiansheng Zheng, Tang Zhu

**Affiliations:** Department of Public Health, School of Basic Medical Science, Putian University, Putian, Fujian 351100, China; Key Laboratory of Translational Tumor Medicine in Fujian Province, School of Basic Medical Science, Putian University, Putian, Fujian 351100, China

**Keywords:** fucosyltransferase 3 (*FUT3*), genotype, inflammatory bowel disease (IBD), meta-analysis, polymorphism

## Abstract

**Background:**

Inflammatory bowel disease (IBD) is a condition with an unclear genetic basis. Fucosyltransferase 3 (FUT3) could potentially be linked to IBD susceptibility.

**Objective:**

To investigate the association between *FUT3* gene polymorphisms and IBD.

**Methods:**

Following the Preferred Reporting Items for Systematic Reviews and Meta-Analyses (PRISMA) 2020 checklist and Population, Intervention, Comparison, Outcomes, and Study (PICOS) guidelines, case-control studies published until April 30, 2020 was searched. Two independent reviewers conducted screening, data extraction, and quality assessment using the Newcastle-Ottawa Scale. Meta-analysis, sensitivity analysis, and Egger tests were performed using RevMan and Stata12.0.

**Results:**

The review included 5 articles and 12 case-control studies involving 1712 IBD patients and 1903 controls. The meta-analysis revealed the following combined odds ratios [95% confidence intervals]: *rs3745635* genotype (*GA+AA vs GG*) 0.84 (0.72–0.97), (*GG+GA vs AA*) 1.93 (1.23–3.05), (*GG vs AA*) 2.38 (1.52–3.74), (*A vs G*) 0.84 (0.73–0.96); *rs3894326* genotype (*TA+AA vs TT*) 1.03 (0.87–1.23), (*TT+TA vs AA*) 1.19 (0.56–2.51), (*TT vs AA*) 1.19 (0.56–2.51), (*A vs T*) 1.02 (0.86–1.20); *rs28362459* genotype (*TG+GG vs TT*) 0.98 (0.85–1.12), (*TT+TG vs GG*) 1.20 (0.90–1.61), (*TT vs GG*) 1.21 (0.90–1.62), (*G vs T*) 0.96 (0.86–1.07). Sensitivity analysis indicated the stability of the results, and Egger analysis showed no significant publication bias.

**Conclusions:**

The *rs3745635* gene polymorphism may be associated with IBD susceptibility, whereas the *rs3894326* and *rs28362459* gene polymorphisms may not be associated with IBD.

Inflammatory bowel disease (IBD) is an idiopathic intestinal inflammatory disease related to environmental, genetic, infections, and immune factors, which can involve the ileum, rectum, and colon [1, 2]. IBD includes Crohn disease (CD) and ulcerative colitis (UC) [3]. The incidence rate of CD and UC is higher in Europe and North America, reaching 14.6–17.4/10 million and 7.6–14.3/10 million populations, respectively. The incidence of CD and UC (0.848 and 1.0/10 million) in China is remarkably lower than that in Europe and North America. However, the incidence rate of IBD clearly increased in the past 2 decades in China [4, 5]. Undoubtedly, genetic factors play an important role in the pathogenesis of IBD [6]. However, more details of the genetic etiology and pathogenesis of IBD, which is prone to chronic or repeated recurrence, have not yet been fully uncovered, resulting in difficulties for clinical prevention and treatment [7].

Human blood group antigen (or Lewis antigen) is a kind of fucosylated protein that is expressed and secreted in the intestinal mucosa [8]. Its expression is catalyzed by fucosyltransferases (FUTs) [9]. So far, at least 13 kinds of FUTs have been confirmed in human beings, the physiological activities of which are not exactly similar [10]. Studies on the FUTs and corresponding disorders are deepening. For instance, association between the polymorphism of the *FUT3* gene and *Toxoplasma gondii* infection [11], breast cancer [12], gastric cancer [13], liver cancer [14], and other diseases has been reported. There have been many studies on the association between polymorphism of the *FUT2* gene and IBD [15,16,17], but corresponding data have not been studied by meta-analysis yet [18]. *FUT3*, which participates in the synthesis of the α-1,3/4 fucoside bond, is one of the crucial enzymes involving Lewis A antigen to determine the composition of intestinal flora. Therefore, it may be associated with IBD susceptibility as well [19]. However, reports concerning the association between the polymorphism of the *FUT3* gene and IBD are limited and in disaccord, without a statistical conclusion [20, 21]. Therefore, this study collected relevant literatures and analyzed the relationship between polymorphism of the *FUT3* genes at *rs3745635*, *rs3894326*, and *rs28362459* and IBD with a meta-analysis, in order to further explore the possible mechanism of the polymorphism of the *FUT3* gene in IBD.

## Methods

This systematic review and meta-analysis was conducted according to the Preferred Reporting Items for Systematic Reviews and Meta-Analyses (PRISMA) 2020 checklist [22]. The study protocol was registered on the International Platform of Registered Systematic Review and Meta-analysis Protocols (INPLASY) database (registration number: 202230001) and is available on inplasy.com (https://doi.org/10.37766/inplasy2022.3.0001) [23]. The research question was “Is fucosyltransferase 3 gene (*rs3745635*, *rs3894326*, *rs28362459*, etc.) an important risk factor for the onset of inflammatory bowel disease?” This research question was constructed following the Population, Intervention, Comparison, Outcomes and Study (PICOS) guidelines[24].

### Data source

“Inflammatory bowel disease, IBD, Crohn* disease, ulcerative colitis, CD, UC, fucosyltransferase3, *FUT3*, *rs3745635*, *G508A*, *rs3894326*, *T1067A*, *rs28362459*, and *T59G*” were applied as keywords to search the corresponding English literature from the PubMed database. Chinese literatures were searched from the China National Knowledge Infrastructure (CNKI), Wanfang, and China Science and Technology Journal (Weipu) databases using “inflammatory bowel disease, Crohn's disease, ulcerative colitis, *FUT3*, fucosyltransferase 3” in Chinese as the keywords.

Taking PubMed as an example, the specific search strategy was as follows: searching all data on (“Inflammatory bowel disease” OR IBD OR “Crohn* disease” OR “Ulcerative colitis” OR CD OR UC) AND (Fucosyltransferase3 OR *FUT3* OR *rs3745635* OR *G508A* OR *rs3894326* OR *T1067A* OR *rs28362459* OR *T59G*). All study searches were completed by April 30, 2020.

### Literature screening

Two literature reviewers independently conducted preliminary screening, rescreening, data extraction, and cross-checking of the retrieved documents. Once divergent results occurred from the 2 reviewers, a third reviewer was invited to assist by judging whether or not to accept the article.

Inclusion criteria were as follows: the study design belonged to the case–control study format; the case group was patients with clinical diagnosis of IBD disease (UC or CD patients), and the control group contained healthy people or other patients without IBD diseases, in addition to both groups of the research subjects being comparable through baseline comparison; phenotype was in line with Hardy–Weinberg balance; the content of the literature included the correlative study of polymorphism of the *FUT3* gene and IBD; the literature data were sufficient to calculate the odds ratio (OR).

The exclusion criteria were as follows: literature was in languages other than Chinese and English; literature was repeatedly published by the same author in different journals; literature contained incomplete data; literature did not meet the requirements; the research object was nonhuman.

### Data extraction and risk assessment of bias

The literature reviewers independently extracted data information from the included literature, including the following: title of the study, first author, published journal, publication time, etc.; baseline information and sample size; results of the *FUT3* gene polymorphism determination; relevant information of the Newcastle–Ottawa Scale (NOS) bias risk assessment [25]. Two reviewers evaluated the quality of each document by using the NOS bias risk assessment standard. If there were different results, the final decision was reached based on consensus after the 2 sides resolved the differences. If the 2 sides still had disputes, a third reviewer was invited to participate in the discussion and decide.

### Study selection

**Figure 1.**
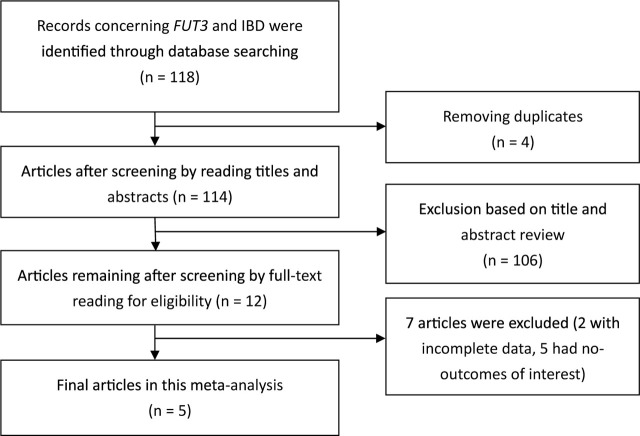
Study selection flow diagram describing the screening process. *FUT3*, fucosyltransferase 3 gene; IBD, inflammatory bowel disease.

Related documents (n = 118) were obtained from the preliminary search. Ultimately, 5 documents were included for the meta-analysis after further screening. These 5 valid literatures included a total of 1712 patients with IBD (1165 patients with UC and 547 patients with CD) and 1903 controls. All the objects met the inclusion criteria. The process of the study selection is shown in **Figure 1**.

### Basic characteristics and quality evaluation of the included literatures

All the included literatures were case–control studies. The basic characteristics of each article are summarized in **Table 1**. The quality evaluation of the included studies was performed by NOS (**Table 2**).

**Table 1.** Basic characteristics of the included manuscripts

**Article**	**Nation**	**Disease**	**IBD**			**Controls**			**Gene**
	
			**Age (years)**	**M:F**	**n**	**Age (years)**	**M:F**	**n**	
Hu et al., 2014 [26]	China	CD	36.24 ± 14.52	121:152	273	36.76 ± 15.62	223:256	479	*rs3745635, rs3894326, rs28362459*
Xu et al., 2014 [27]	China	UC	42.25 ± 13.28	130:103	233	40.96 ± 15.42	157:135	292	*rs3745635, rs3894326, rs28362459*
Guo et al., 2015 [28]	China	UC	41.56 ± 14.45	224:165	389	41.24 ± 16.01	263:229	492	*rs3745635, rs3894326, rs28362459*
	China	CD	39.49 ± 16.51	153:121	274				
Hu et al., 2016 [29]	China	UC	40.23 ± 15.32	201:284	485	41.09 ± 17.11	261:319	580	*rs3745635, rs3894326, rs28362459*
Chen et al., 2018 [30]	China	UC	36–59	31:27	58	34–60	34:26	60	*rs3745635, rs3894326, rs28362459*

CD, Crohn disease; IBD, inflammatory bowel disease; UC, ulcerative colitis.

**Table 2.** Quality evaluation of the included articles with NOS

**Article**	**Item 1**	**Item 2**	**Item 3**	**Item 4**	**Item 5**	**Item 6**	**Item 7**	**Item 8**	**Score**
Hu et al., 2014 [26]	Yes	Unclear	Yes	Yes	Age, gender	Yes	Yes	Yes	8
Xu et al., 2014 [27]	Yes	Unclear	Yes	Yes	Age, gender	Yes	Yes	Yes	8
Guo et al., 2015 [28]	Yes	Unclear	Yes	Yes	Age, gender	Yes	Yes	Yes	8
Hu et al., 2016 [29]	Yes	Unclear	Yes	Yes	Age, gender	Yes	Yes	Yes	8
Chen et al., 2018 [30]	Yes	Unclear	Yes	Yes	Age, gender	Yes	Yes	Yes	8

Item 1: Is the case definition adequate?

Item 2: Representativeness of the cases.

Item 3: Selection of controls.

Item 4: Definition of controls.

Item 5: Comparability of cases and controls on the basis of the design or analysis (1 = age; 2 = gender).

Item 6: Ascertainment of exposure.

Item 7: Same method of ascertainment for cases and controls.

Item 8: Nonresponse rate.

NOS, Newcastle–Ottawa Quality Assessment Scale.

### Statistical analysis

RevMan 5.3 software [31] was applied for meta-analysis of the dominant gene model, recessive gene model, codominant gene model, and allele frequency of *FUT3*. Heterogeneity was tested by heterogeneity (α = 0.1) and *I*^2^ quantitative judgment method. If the heterogeneity test *P* > 0.1 and *I*^2^ <50%, a fixed-effect model would be used for the meta-analysis. If the heterogeneity test *P* ≤ 0.1 or *I*^2^ ≥50%, a random-effect model was applied for the meta-analysis after excluding the effects of obvious clinical heterogeneity. The OR and 95% confidence interval (CI) effect size were used as indicators of binary qualitative variable data. Sensitivity analysis was performed by eliminating each original study one by one. Stata12.0 software was applied for Egger test to analyze the publication bias. The inspection level α = 0.05.

## Results

### Association of the *rs3745635* polymorphism with IBD

**Figure 2.**
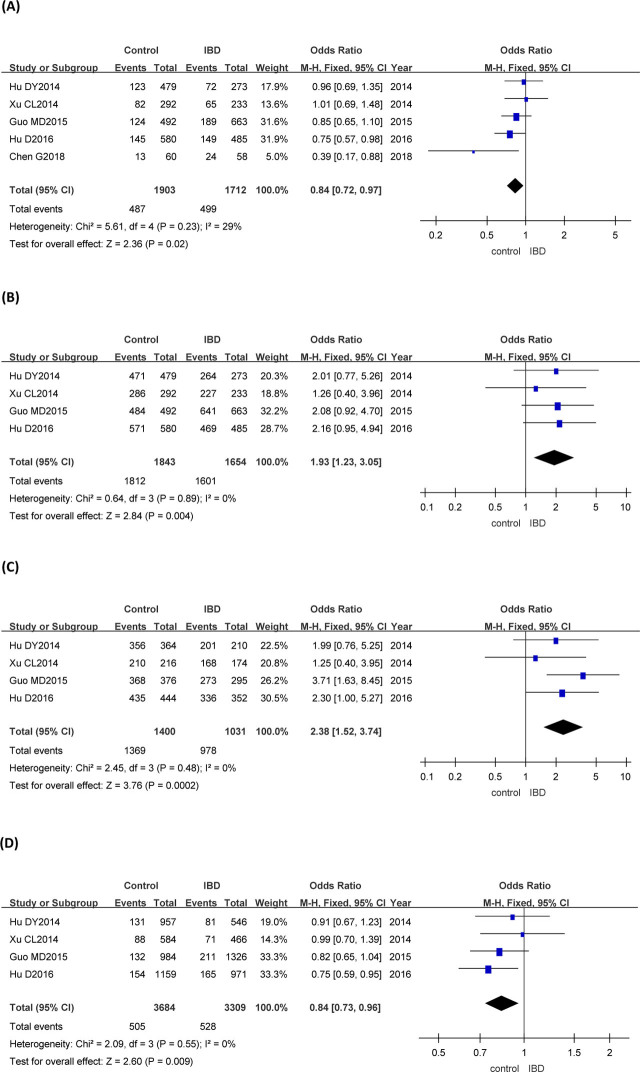
Forest plots of meta-analysis of the genotypes of *rs3745635* and IBD. The odds ratios and 95% CIs of the 1712 patients with IBD with *GA* + *AA* vs *GG rs3745635* genotype and the 1903 controls from 5 articles (**A**), the 1654 patients with IBD with *GG* + *GA* vs *AA rs3745635* genotype and the 1843 controls from 4 articles (**B**), the 1031 patients with IBD with *GG* vs *AA rs3745635* genotype and the 1400 controls from 4 articles (**C**), and the 3309 patients with IBD with *A* vs *G rs3745635* genotype and the 3684 controls from 4 articles (**D**) were analyzed by the M–H fixed-effect model. CI, confidence interval; IBD, inflammatory bowel disease; M–H, Mantel–Haenszel.

*GA* + *AA* vs *GG* genotype of the *rs3745635* gene was studied in 5 articles. *GG* + *GA* vs *AA*, *GG* vs *AA*, and *A* vs *G* genotypes of the *rs3745635* gene were explored in 4 articles. Meta-analysis of the fixed-effect model showed that there was statistical significance in *rs3745635* dominant genotype, recessive genotype, codominant genotype, and allele frequency between IBD group and control group (**Figure 2** and **Table 3**).

**Table 3.** Meta-analysis of the association between *FUT3* and IBD

**Gene**	**Genetic model**	**Heterogeneity**			**Consolidated results**			**Egger test**	

		***χ* ^2^**	***P***	***I*^2^ (%)**	**OR (95% CI)**	***Z***	***P***	***t***	***P***
*rs3745635*	*GG* + *GA* vs AA	0.64	0.89	0	1.93 (1.23–3.05)	2.84	0.004	1.65	0.242
*rs3745635*	*GA* + *AA* vs GG	5.61	0.23	29	0.84 (0.72–0.97)	2.36	0.02	0.87	0.447
*rs3745635*	*GG* vs *AA*	2.45	0.48	0	2.38 (1.52–3.74)	3.76	0.000	0.15	0.897
*rs3745635*	*A* vs *G*	2.09	0.55	0	0.84 (0.73–0.96)	2.60	0.009	3.55	0.071
*rs3894326*	*TA* + *AA* vs *TT*	1.79	0.77	0	1.03 (0.87–1.23)	0.39	0.70	0.01	0.990
*rs3894326*	*TT* + *TA* vs *AA*	1.17	0.76	0	1.19 (0.56–2.51)	0.46	0.65	1.31	0.321
*rs3894326*	*TT* vs *AA*	1.24	0.74	0	1.19 (0.56–2.51)	0.45	0.65	1.32	0.317
*rs3894326*	*A* vs *T*	1.96	0.58	0	1.02 (0.86–1.20)	0.22	0.83	0.69	0.563
*rs28362459*	*TG* + *GG* vs *TT*	2.25	0.69	0	0.98 (0.85–1.12)	0.35	0.73	0.68	0.546
*rs28362459*	*TT* + *TG* vs *GG*	0.10	0.99	0	1.20 (0.90–1.61)	1.25	0.21	1.00	0.424
*rs28362459*	*TT* vs *GG*	0.26	0.97	0	1.21 (0.90–1.62)	1.25	0.21	0.37	0.746
*rs28362459*	*G* vs *T*	1.67	0.64	0	0.96 (0.86–1.07)	0.78	0.44	0.34	0.764

CI, confidence interval; *FUT3,* fucosyltransferase gene; IBD, inflammatory bowel disease; OR, odds ratio.

### Association of the *rs3894326* polymorphism with IBD

**Figure 3.**
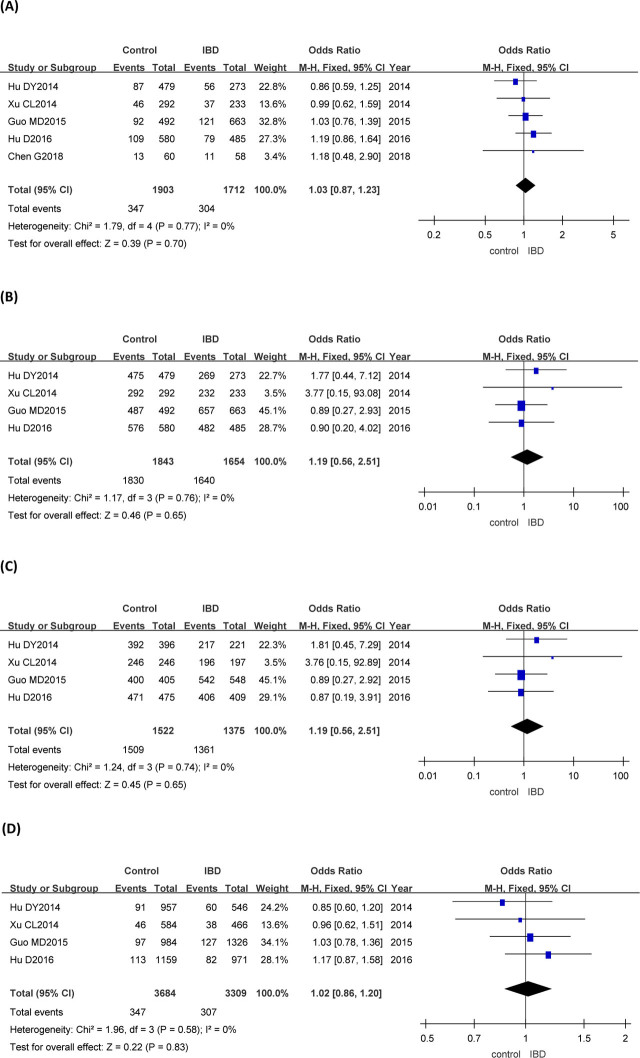
Forest plots of meta-analysis of the genotypes of *rs3894326* and IBD. The odds ratios and 95% CIs of 1712 patients with IBD with *TA* +*AA* vs *TT* genotype of the *rs3894326* gene and the 1903 controls from 5 articles (**A**), the 1654 patients with IBD with *TT* + *TA* vs *AA* genotype of the *rs3894326* gene and the 1843 controls from 4 articles (**B**), the 1375 cases of IBD with *TT* vs *AA* genotype of the *rs3894326* gene and the 1522 controls from 4 articles (**C**), and the 3309 patients with IBD with *A* vs *T* genotype of the *rs3894326* gene and the 3684 controls from 4 articles (**D**) were analyzed by the M–H fixed-effect model. CI, confidence interval; IBD, inflammatory bowel disease; M–H, Mantel–Haenszel.

*TA* + *AA* vs *TT* genotype of the *rs3894326* gene was studied in 5 articles. *TT* + *TA* vs *AA*, *TT* vs *AA*, and *A* vs *T* genotypes of the *rs3894326* gene were explored in 4 articles. The fixed-effects model used in the meta-analysis showed that there was no statistical significance in *rs3894326* dominant genotype, recessive genotype, codominant genotype, and allele frequency between IBD group and control group (**Figure 3** and **Table 3**).

### Association of the *rs28362459* polymorphism with IBD

**Figure 4.**
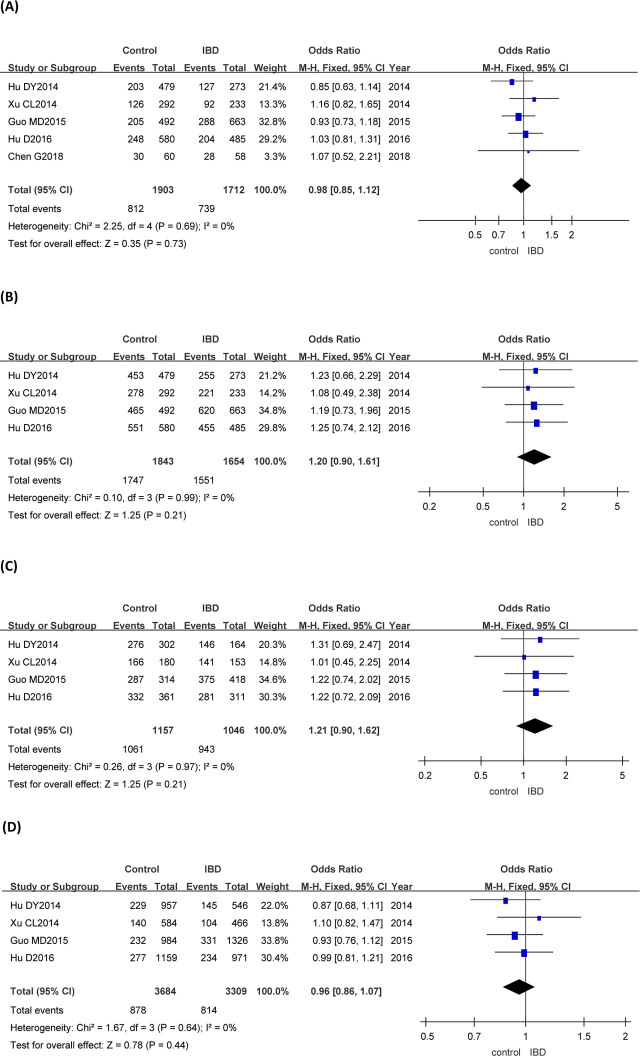
Forest plots of meta-analysis of the genotype of the *rs28362459* gene and IBD. The odds ratios and 95% CIs of the 1712 cases of IBD with *TG* + *GG* vs *TT* genotype of the *rs28362459* gene and the 1903 controls from 5 articles **(A)**, the 1654 patients with IBD with *TT* + *TG* vs *GG* genotype of the *rs28362459* gene and the 1843 controls from 4 articles **(B)**, the 1046 patients with IBD with *TT* vs *GG* genotype of the *rs28362459* gene and the 1157 controls from 4 articles **(C)**, and the 3309 patients with IBD with *G* vs *T* genotype of the *rs28362459* gene and the 3684 controls from 4 articles **(D)** were analyzed by the M–H fixed-effect model. CI, confidence interval; IBD, inflammatory bowel disease; M–H, Mantel–Haenszel.

*TG* + *GG* vs *TT* genotype of the *rs28362459* gene was studied in 5 groups. *TT* + *TG* vs *GG, TT* vs *GG, G* vs *T* genotypes of the *rs28362459* gene were analyzed in 4 articles. Meta-analysis of the fixed-effect model (M–H) showed that there was no statistical significance in *rs28362459* dominant genotype, recessive genotype, codominant genotype, and allele frequency between IBD group and control group (**Figure 4** and **Table 3**).

### Sensitivity analysis

In the meta-analysis of the *GA* + *AA* vs *GG* and *A* vs *G* genotypes of the *rs3745635* gene, the conclusion of the combined OR value after excluding Hu's research data [26] was inconsistent with that before the elimination. For the remaining other 10 genotypes, the conclusion of the combined OR value after excluding the original research data in turn was consistent with the conclusion before the exclusion.

### Publication bias analysis

Since the number of documents included in the meta-analysis of the association of the polymorphisms of the *FUT3* genes with IBD was <10, it was not appropriate to perform funnel chart analysis. Egger linear regression analysis using STATA12.0 was then applied, which showed that all genotypes considered for Egger linear regression analysis in the meta-analysis were not statistically significant (**Table 3**).

## Discussion

*FUT3* is a gene located in the *19q13* region of the human chromosome. We noticed that all the 5 papers concerned Chinese populations. There were few studies on the association between the polymorphism of the *FUT3* gene and IBD in other populations. The most common functional loci of the *FUT3* gene in the Chinese population were *rs3745635*, *rs3894326*, and *rs28362459* [32]. So far, studies on the association of the polymorphism of the *FUT3* gene with IBD are quite limited globally. Based on strict literature evaluations, this study collected 5 case–control studies and analyzed the correlation between the polymorphism of the *FUT3* gene and IBD. It avoided the impact of small sample size and single study error and relatively objectively and comprehensively evaluated the association of the polymorphism of the *FUT3* gene with IBD.

Lewis antigen in the intestine is an adhesion receptor, which can not only mediate the adhesion and binding of various pathogenic microorganisms in the gastrointestinal tract, such as *Helicobacter pylori*, norovirus, *Campylobacter jejuni*, and so on [33], but can also decompose into fucose, galactose, and other substances under the action of α-glucosidase, providing a carbon source for the metabolic activities of normal intestinal flora [19, 34]. All the *rs3745635*, *rs3894326*, and *rs28362459* genotypes of the *FUT3* gene are related to the synthesis of α-1,3/4 fucoside bond, which is one of the key enzymes involved in LewisA antigen and determines fucosylation. The α-1,3/4 fucoside bond is crucial for the maturation and function of the Lewis antigen [10]. Any mutations of the *rs3745635* and *rs3894326* functional site will cause loss of activity of α-1,3/4 fucosyltransferase, resulting in dysfunction of the intestinal Lewis antigen and its depletion [35]. However, mutation at the *rs28362459* functional site will alter the spatial conformation of the transmembrane region of the α-1,3/4 fucosyltransferase, thus reducing the intestinal Lewis antigen expression level [33].

In this study, the *GA* + *AA* vs *GG*, *GG* + *GA* vs *AA*, *GG* vs *AA*, and *A* vs *G* genotypes of the *rs3745635* gene were analyzed by systematic evaluation. The combined OR values (95% CIs) were 0.84 (0.72–0.97), 1.93 (1.23–3.05), 2.38 (1.52–3.74), and 0.84 (0.73–0.96), respectively, indicating that all the dominant gene model, recessive gene model, codominant gene model, and allele frequency of the *rs3745635* were associated with IBD. The possible reason is that *rs3745635* mutation of the *FUT3* gene may lead to abnormal levels of the Lewis antigen in the intestine, which may lead to imbalance of intestinal flora, disorder of intestinal mucosal immune regulation, and inflammatory injury of intestinal tissue, thus affecting the susceptibility to IBD. Theoretically, mutation of the *rs3894326* and *rs28362459* genotypes of the *FUT3* gene may lead to abnormal levels of the Lewis antigen in the intestinal tract as well. However, our analysis showed that the dominant gene model, recessive gene model, codominant gene model, and allele frequency of the *rs3894326* and *rs28362459* loci were not significantly correlated with IBD. As the data from Nakashima et al. [11] show, the *rs28362459* mutation only affects the conformation of the α-1,3/4 fucosyltransferase, relatively reducing the intestinal level of Lewis antigen. This alteration of the α-1,3/4 fucosyltransferase may not affect the intestinal flora distribution enough. Thus, the mutation of the *rs28362459* is not associated with IBD. However, similar to *rs3745635*, the mutation of the *rs3894326* is reported to reduce the activity of α-1,3/4 fucosyltransferase and prevent the expression of intestinal Lewis antigen [35]. However, in our study, it still was not statistically associated with IBD. The possible explanation could be that the pathology of IBD is a complex process, which involves not only genetic factors but also other factors, such as environment, infection, and immunity as well. There may be some other unknown factors involved in the pathological process of the *rs3894326* mutation–induced IBD.

In this study, all the *P* values and *I*^2^ results of the heterogeneity test for the included literatures were >0.1 and <50%, respectively. It indicated that there was no serious heterogeneity in the cited articles; thus, the research literatures could be merged. No significant difference was found in Egger linear regression analysis for all genotypes and IBD in the meta-analysis, suggesting that there was no significant publication bias. However, there were still some shortcomings. Firstly, all the study subjects in the included literatures were Chinese populations. The conclusion from this study may not be extended to other populations since the genetic information in other populations is different. Whether this mutation of the *rs3745635* genotype of *FUT3* is associated with IBD in other populations will need to be confirmed further. Secondly, the number of research papers and the samples are still small. Thirdly, sensitivity analysis of the *GA* + *AA* vs *GG* and *A* vs *G* of the r*s3745635* genotype showed that their conclusions were inconsistent, indicating that the conclusions were not stable enough. Finally, the included literatures did not show other possible influencing factors, such as age, gender, smoking, drinking, and other factors, concerning the disease, nor did they investigate the interaction between genes, let alone the interaction between the environment and the genes.

## Conclusion

All the dominant gene model, recessive gene model, codominant gene model, and allele frequency of the *rs3745635* locus were associated with IBD. The dominant gene model, recessive gene model, codominant gene model, and allele frequency of the *rs3894326* and *rs28362459* loci may not be associated with IBD. IBD is an idiopathic intestinal inflammatory disease related to environmental, genetic, infections, and immune factors. Further research is still needed in the future.
